# Prevalence of onychomycosis among psoriasis patients: a clinico-mycological and dermoscopic comparative cross sectional study

**DOI:** 10.1038/s41598-024-71321-6

**Published:** 2024-09-18

**Authors:** Hamed M. Abdo, Hussein M. Hassab-El-Naby, Mohamed R. Bashtar, Mohamed S. Hasan, Mohamed L. Elsaie

**Affiliations:** 1 Department of Dermatology, Venereology and Andrology, Faculty of Medicine, Al-Azhar University, Cairo, Egypt; 2 Department of Dermatology, Venereology and Andrology, Medical Research and Clinical Studies Institute, National Research Centre, Giza, Egypt

**Keywords:** Psoriasis, Oychomycosis, Mycology, Dermoscopy, Histopathology, Diseases, Health care, Medical research

## Abstract

Onychomycosis, a nail infection caused by dermatophytes, yeast, and molds makes up roughly half of all onychopathies and is the most prevalent nail condition in the world. Clinically, nail psoriasis and onychomycosis can frequently be difficult to distinguish from one another. To assess the prevalence of onychomycosis in patients with psoriasis. Fifty patients with psoriasis associated with nail disease were included in this study. After taking clinical history, nail samples were gathered for dermoscopic inspection, culture, direct microscopy with 20% KOH solution, and nail clipping with PAS stain. Of the 50 patients recruited, 43 were males and 7 were females, with mean age 6–71 years (mean ± SD 44.06 ± 16.2). Eleven patients (22%) tested positive for onychomycosis. Dermatophytes were isolated from 2% of patients, yeast from 14% of patients, and non-dermatophytic mold from 38% of patients. Histopathological results revealed fungal hyphae and spores in 18% of patients. The most prevalent dermoscopic sign in psoriatic patients with onychomycosis was spikes (81.8%) with statistical significance (P-value < 0.001), while nail pitting was the most prevalent dermoscopic feature in nail psoriasis. This study lays the way for an accurate diagnosis of nail lesions by highlighting the significance of cooperation between mycology, histology, and dermoscopy in the diagnosis of onychomycosis in patients with nail psoriasis.

## Introduction

Psoriasis is characterized clinically by skin lesions and extracutaneous comorbidities side by side to systemic inflammation^1^. Erythematous, indurated, and scaling plaques with discomfort, itching, and a burning feeling are typical cutaneous signs of psoriasis^2^.

A fungal infection of the nail unit, specifically the nail bed, known as onychomycosis results in gradual changes to the nail's color, texture, and structure. Regardless of the cause, it is an invasive nail fungal infection^3^. It has an insidious onset and if left untreated, progresses until it involves the entire nail plate. Onychomycosis rarely resolves spontaneously, nor does it respond to placebo therapy taken orally or applied topically^3^.

Onychomycosis is more common in people with nail psoriasis^4^. According to studies, immunosuppressive medications and/or structural changes in nail psoriasis may be the main causes^5,6^. Onychomycosis was not considerably more common in psoriatic patients, according to some experts. The rapid growth of the affected nails in psoriasis may inhibit the development of onychomycosis due to the quick turnover and elimination of the distal nail plate, possibly decreasing the opportunity for fungi to invade the nail keratin^7^. This is one explanation for why psoriasis may increase nail protection against fungal invasion^8–10^. Additionally, the serum like glycoprotein substance that was discovered in psoriatic oil drop patches may possibly have an inhibitory effect on dermatophytes^11^. This study included mycological, histopathological, and dermoscopical examinations to assess the prevalence of onychomycosis in psoriasis patients.

## Patients and methods

Fifty psoriatic patients (diagnosed clinically or pathologically) associated with finger nail disease were included in this study. Participants were recruited from the outpatient clinic of the Dermatology and Venerology department at Al-Azhar University Hospital in Cairo, Egypt. The study was approved by the Faculty of Medicine Al-Azhar University's Research Ethics Committee (00012367-21-02-002). All participants or their guardians gave their informed consent to participate in the trial. All patients of the study were subjected to detailed history-taking including personal history, history of the present illness, past history, and family history of psoriasis. Patients who received any topical or systemic antifungals during the previous 3 months or were suffering from any disease-causing nail dystrophy such as eczema and lichen planus were excluded.

### Mycological examination

The suspected nails were cleaned with 70% alcohol to remove contaminants. Scrapings were taken with a sterile scalpel blade and collected in a sterile clean container. The friable subungual debris was collected as well as the scraping of the nail bed. The collected specimens were divided into three portions. The first portion of the specimens was examined microscopically using 20% potassium hydroxide (KOH). The second and third portions were cultured on Sabouraud dextrose agar, one with and another without cycloheximide.

### Histopathological examination

After collection of the specimens for mycological testing, a nail clipping is taken and put in a plane tube. With the help of a nipper, a fragment of at least 5 mm longitudinally and 2 mm transversely of the affected nails was cut for an adequate fragment fixation in paraffin. The extracted specimen was placed in a formaldehyde solution. When processing this specimen, a chitin-softening or Tween solution may be used. Periodic acid Schiff (PAS) stain for the demonstration of fungal hyphae was used in the current study.

### Onychoscopy

All affected nails were examined by a handheld dermatoscopy (DermliteDL4N; 4 Gen, Inc, San Juan Capistrano, CA), with a magnification of 10×. Higher magnification of up to 30× was used wherever deemed necessary. Images were recorded directly by a device Samsung Note 10 Plus attached to the dermoscopy. Both non-polarized and polarized modes were used. Non-polarized and dry mode helps in the detection of nail plate changes, whereas polarized mode with or without fluid is best for nail bed changes.

Based on clinical, mycology, histopathology and dermoscopy results (CMHD), we have postulated that presence of a clinical picture including (subungual hyperkeratosis, onycholysis, nail discoloration) as well fungal elements in histopathology with dermatophytes in culture as well as key dermoscopic features of (spikes, ruin appearance, white lunula and aurora patterns) to be a definite diagnosis of onychomycosis.

## Results

The age of the patients ranged from 6 to 71 years, with a mean of 44.06 ± 16.2, including 7 (14%) females and 43 (86%) males. The time of diagnosis of psoriatic disease ranged from 1 to 40 years previously among patients with a mean of 11.2 ± 7.9 years. Table 1 Regarding the manifestations, the most common type of psoriasis was plaque psoriasis (n = 27; 54%), and all patients were on topical treatments and the most common systemic treatment used was methotrexate.

**Table 1:** Demographic data in all studied patients.

Data		Studied patients (N = 50)	
Age (years)	Mean ± SD	44.06 ± 16.2	
	Min–Max	6–71	
Sex	Male	43	86%
	Female	7	14%
Occupation	Manual worker	21	42%
	Non-manual worker	29	58%
Risk factors	Smoking	12	24%
	Sun exposure	34	68%
Family history	Negative	39	78%
	Positive	11	22%
Co-morbidities	Psoriatic arthritis	18	36%
	HTN	6	12%
	DM	6	12%
Residence	Rural	33	66%
	Urban	17	34%

DM: diabetes mellitus; HTN: hypertension.

**Fig. 1:**
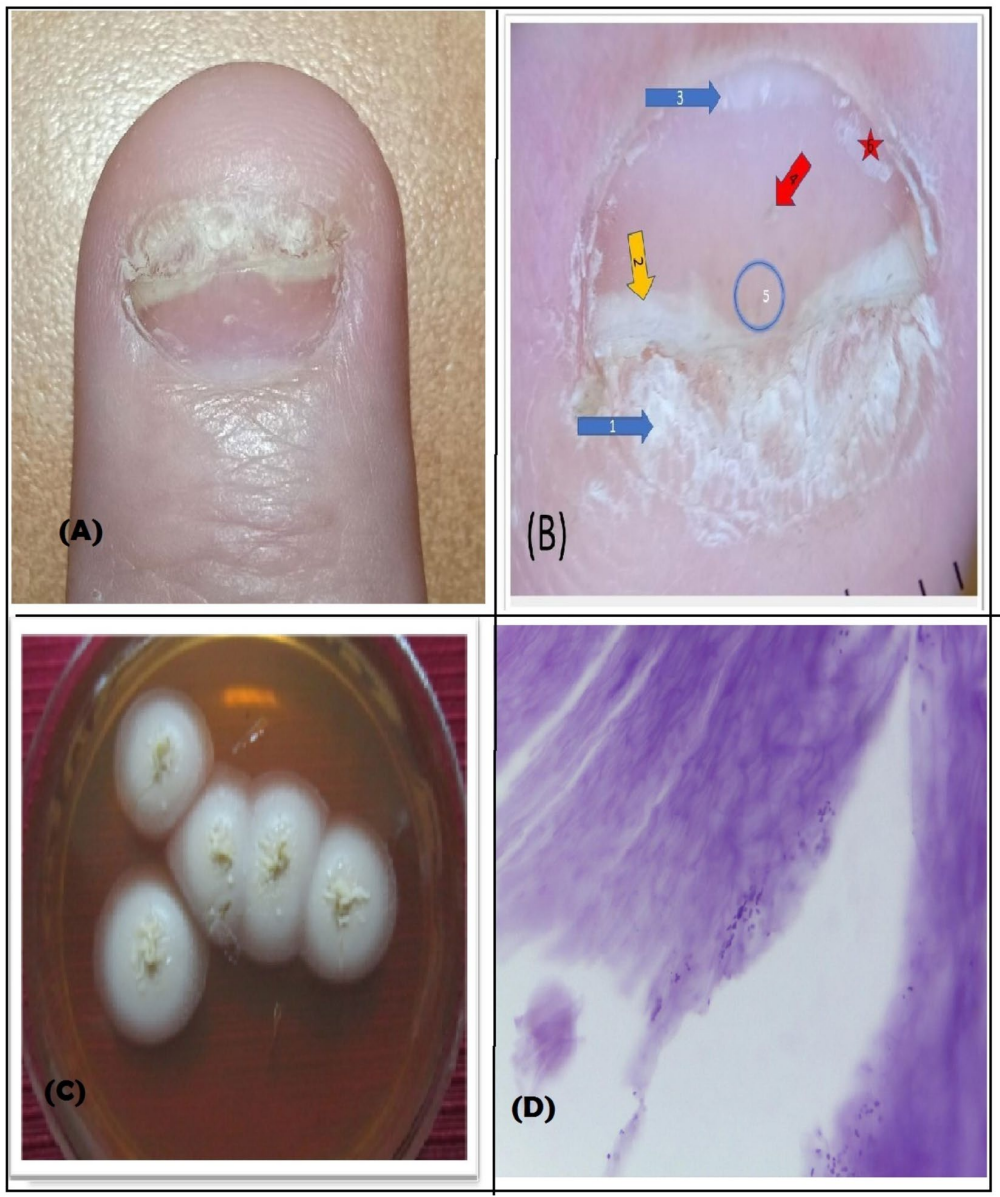
(**A**) clinical photo shows: DLSO. (**B**) dermoscopy photo shows: 1 = subungual hyperkeratosis, 2 = salmon patch, 3 = structureless area (onycholysis), 4 = pitting. (**C**) mycology culture shows trichosporon species, (**D**) KOH examination shows trichosporon species, and (**E**) histopathology photo shows: fungal spores.

**Fig 2.**
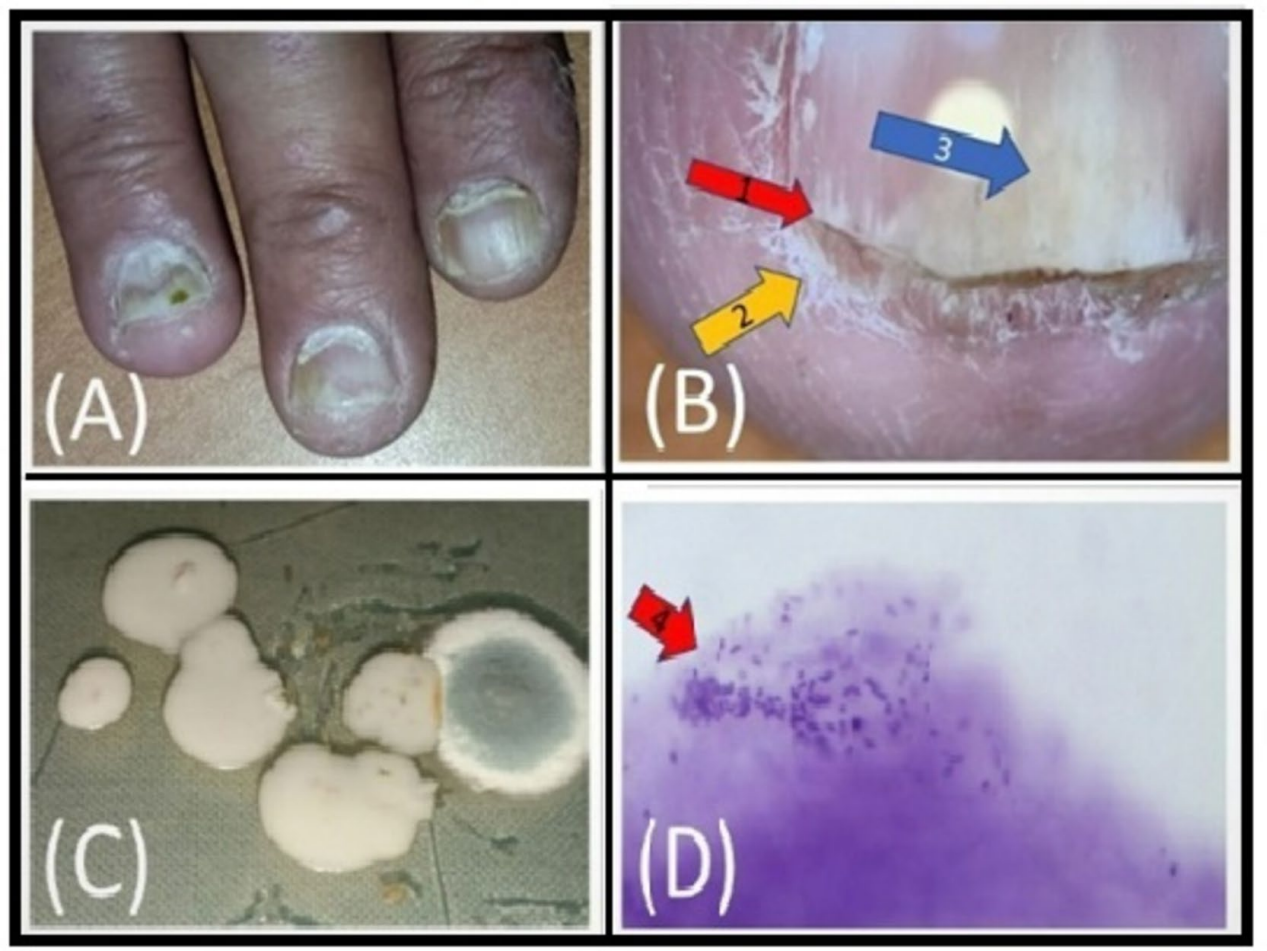
(**A**) clinical photo shows: DLSO. (**B**) dermoscopy photo shows: 1 = spikes, 2 = subungual hyperkeratosis, 3 = salmon patch. (**C**) mycology culture shows Candida species. (**D**) Histopathology photo shows: 4 = fungal spores.

Mycological analyses showed among the 50 patients, the rate of onychomycosis to be 22% (11 patients). The least frequent fungi were from the dermatophyte group (one patient, 2%), and identified as *T. Violaceum*. Yeasts were identified in seven cases (14%), and all occurred on the fingernails but three simultaneously on fingernails and toenails. *C. parapsilosis* was the most frequent species (five patients, 10%). There was growth of nondermatophyte fungi in nineteen (38%) of culture of which *Aspergillus* and *Penicillium* species were the commonest (Tables 2, 3, 4; Figs. 1, 2).

**Table 2:** Isolated fungi.

Isolated fungi	Studied group N = 50	
Dermatophytes; No. = 1 (2%)		
* Trichophyton violaceum*	1	2%
Yeasts; No. = 7 (14%)		
* Candida parapsilosis*	5	10%
* Trichosporon species*	2	4%
Non-dermatophytic molds; No. = 19 (38%)		
Single growth No. = 11 (22%)		
* Aspergillus species*	3	6%
* Penicillium species*	2	4%
* Unknown molds*	2	4%
* Alternaria species*	1	2%
* Cladosporium species*	1	2%
* Phialophora species*	1	2%
* Fonsecaea species*	1	2%
Mixed growth; No. = 8 (16%)		
Mold + mold; No. = 6 (12%)	6	12%
Mold + yeast; No. = 2(4%)	2	4%
Histopathology results		
Positive	9	18%
Negative	41	82%

**Table 3:** Correlation between diagnosis of onychomycosis and dermoscopic results in all studied patients.

		Diagnosis				X^2^	P-value
		Psoriasis without onychomycosis (N = 39)		Psoriasis with onychomycosis (N = 11)			
Dermoscopy	Pitting	35	89.7%	6	54.5%	7.2	0.007 S
	Splinter hemorrhage	18	46.2%	5	45.4%	0.001	0.967 NS
	Ruin pattern	0	0%	7	63.6%	28.8	< 0.001 HS
	Onycholysis	31	79.4%	5	45.5%	4.9	0.026 S
	Subungual hyperkeratosis	12	30.8%	7	63.6%	3.9	0.047 S
	Spikes	0	0.0%	9	81.8%	38.9	< 0.001 HS
	Salmon patch	18	46.2%	5	45.4%	0.001	0.967 NS
	Longitudinal striation	2	5.1%	4	36.4%	7.9	0.005 S
	Transverse striations	7	17.9%	6	54.5%	5.9	0.015 S
	Nail thickening	0	0%	6	54.5%	24.1	< 0.001 HS
	Oil drop	0	0%	0	0%	–	–
	Aurora borealis	0	0%	0	0%	–	–

χ^2^: chi-square test; S significant; HS: highly significant; NS: non-significant.

**Table 4:** Correlation between diagnosis and clinical findings in all studied patients.

		Diagnosis				X^2^	P-value
		Psoriasis with no onychomycosis (N = 39)		Psoriasis with onychomycosis (N = 11)			
Clinical Findings	Subungual hyperkeratosis	12	30.8%	7	63.6%	**3.9**	**0.047 S**
	Onycholysis	31	79.5%	6	54.5%	2.77	0.095 NS
	Nail discoloration (yellow)	8	20.5%	10	90.9%	**18.5**	** < 0.001S**
	Pitting	35	89.7%	5	45.5%	**10.5**	**0.001 S**
	Splinter hemorrhage	10	25.6%	2	18.2%	0.26	0.608 NS
	Oil drop	0	0%	0	0%	–	–
	Leukonychia	5	12.8%	0	0%	1.56	0.210 NS
	Paronychia	1	2.6%	0	0%	0.28	0.591 NS

χ^2^: chi-square test; S: significant; HS: highly significant; NS: non-significant.

Significant values are in bold.

Regarding dermoscopic signs in nail psoriasis without onychomycosis (Table 3), we found that pitting was the most prevalent feature (89.7%). The second most common dermoscopic finding in nail psoriasis was onycholysis (79.4%). Spikes were significantly the most common finding occurring in 9 out of 11 cases (81.8%) (P-value < 0.001) when compared to psoriatic cases without onychomycosis. This was followed by the ruin pattern in 7 cases (63.6%).

The histopathological analysis showed 9 (18%) positive samples for fungi and three (7.9%) showed typical histological alterations of psoriasis. but this does not allow the identification of the fungal species present. regarding dermoscopic signs in psoriatic patients with onychomycosis, spikes were the most significant common finding in 9 out of the 11 (81.8%) patients (P-value < 0.001) (Tables 2, 3, 4).

## Discussion

Psoriasis is a common chronic inflammatory skin disease that affects approximately 2% of the population in the Western world^12^. Onychomycosis is a fungal infection of the nail plate caused by dermatophytes, yeast, and molds. Onychomycosis is the most common nail disease worldwide and constitutes about half of all onychopathies. Both psoriasis and onychomycosis are common diseases in the general population^13^.

In the current study, the patient’s age ranged from 6 to 71 years; with a mean age of (44.06 ± 16.2) that matched other studies showing mid forties to be an average mean age for seeking medical advice in general population^14,15^. Male patients were 43 (86%) and female patients were 7 patients (14%) and the duration of psoriasis ranged between 1 and 40 years with mean of 11.2 ± 7.9 years.

Concerning the associated diseases, 6 patients had hypertension (12%), 6 patients had diabetes (12%) while 18 (36%) suffered from psoriatic arthritis. Similar to our results, several studies found that diabetes mellitus and hypertension are more prevalent in psoriasis patients^16–18^. Aalemi et al., Chiu et al., Duan et al.^17–19^. The current study found that 11 patients (22%) had a family history of psoriasis. A higher incidence was reported by Solmaz et al.^19^ who found that 31.9% of their patients had a family history of psoriasis or psoriatic arthritis while Augustin et al.^20^ found positive family history in 11% of patients with nail psoriasis.

In the current study, eleven out of the 50 psoriatic patients (22%) were diagnosed as having onychomycosis. Histopathological diagnosis was conclusive in nine cases (18%) while mycological cultures demonstrated dermatophyte in one patient and dermoscopic criteria of onychomycosis (ruin pattern and spikes) were present in sixteen patients. A lower prevalence of onychomycosis in patients with nail psoriasis was found by Klaassen et al.^21^, Gupta et al.^22^, and Al-Mutairi et al.^23^, who found a prevalence of 18%, 10.22%, and 20.3% respectively. On the other hand, a higher prevalence was observed by Tabassum et al.^24^, Tsentemeidou et al.^25^, Rigopoulos et al.^8^ who reported the frequency of onychomycosis among patients to be 34%. Other authors (Jendoubi et al.^26^ and Alves et al.^27^), reported onychomycosis prevalence among psoriatic patients to be 53% and 57.89% respectively. The highest incidence (62%) was reported by Zisova et al.^28^ in a military population. The discrepancy in the results of the different studies could be explained by the use of different diagnostic parameters. Obviously, the correlation between mycological, histopathological, and dermoscopic tools as practiced in this study will largely exclude false-positive and false-negative results.

Trichophyton violaceum was the only isolated dermatophyte from one patient (2%) in the current study. This was relatively less than the results of Romaszkiewicz et al.^29^, and Tabassum et al.^24^ who isolated dermatophytes in 7% and 6.6% respectively. Alves et al.^27^ isolated dermatophytes in 23.6% mostly from toenails in Brazil among which half of them belonged to T. rubrum. Generally, mycoses are endemic in countries of South America, especially in rural communities which could explain the increased incidence of onychomycosis in the later study.

Non-dermatophytic molds and yeasts were isolated from 19 (38%) and 7 patients (14%) respectively; of which were identified as *Candida* and *Trichosporon species*. Romaszkiewicz et al.^29^, isolated *Candida species* from 10 patients (9.8%) and *Geotrichum species* from 2 patients (1.96%). Yeasts were isolated with a higher percentage in the studies of Kacar et al.^30^, Tabassum et al.^24^, Alves et al.^27^, and Romaszkiewicz et al.^29^ where the incidence was 23%, 36%, 43%, and 50% respectively. A lower incidence of non-dermatophytic mold isolation was reported by Tabassum et al.^24^, in 36%, and Kacar et al.^30^ in 15%. On the other hand, Alves et al.^24^ reported no isolation of non dermatophyte filamentous fungi. Earlier conclusions identified nails of psoriasis patients are at a higher risk of colonization; as altered subungual tissue and onycholysis could facilitate yeast invasion^31^.

The difference in fungal isolation rates could be related to the varied demographic characteristics of the patients enrolled in the studies. Also, different methods of fungal identification and interpretation between different studies could be a factor. Moreover; the role of immunosuppressive medications in inhibiting non dermatophytes and possibly facilitating proliferation of dermatophytes and yeasts can also be considered when interpreting such findings^31^. Another aspect is the possibility that the use of nail clippings collects samples only from the distal portion of the nail, reducing positivity rates due to the absence of live fungal structures in the distal portion. Increased positivity could be achieved by collecting material from the proximal portion of the nails^32^.

On PAS-stained histopathological nail clippings, fungal hyphae and spores were found in 9 patients (18%) that matched results of Abu El-Hamd et al.^33^ In the current study, the positive histopathological result of fungal elements was considered to be diagnostic of onychomycosis. Yet, still, many researchers depend on mycological cultures and dermoscopic signs because it is easier and less aggressive.

Dermoscopic examination in cases diagnosed with onychomycosis found that spikes were significantly the most common finding occurring in 9 out of 11 cases (81.8%) (P-value < 0.001) when compared to psoriatic cases without onychomycosis. This was followed by the ruin pattern in 7 cases (63.6%) which was in accordance with Ankad et al.^34^ who observed spikes and ruin pattern in 90% and 65% respectively.

Regarding dermoscopic signs in nail psoriasis without onychomycosis (Table 3), we found that pitting was the most prevalent feature (89.7%). Similar results were reported by other studies^35–40^. The highest incidence of pitting (92.5%) was reported by Polat and Kapıcıoğlu^41^. The second most common dermoscopic finding in nail psoriasis was onycholysis (79.4%). Different studies reported onycholysis to be the most common clinical sign of nail psoriasis^36,42,43^. On the other hand, a study by Yorulmaz et al.^44^, found splinter hemorrhage (73.1%) to be the most common clinical finding in nail psoriasis.

The study had some limitations, such as the sample number, which was relatively small to allow broader conclusions. In conclusion; the present study establishes the way for an accurate diagnosis of nail lesions by highlighting the significance of utilizing mycology, histology, and dermoscopy in the diagnosis of onychomycosis in patients with nail psoriasis. Further investigations are needed, including longitudinal and randomised studies to obtain further evidence on the relationship between onychomycosis and psoriatic disease.

## Data Availability

"The data that support the findings of this study are available from the corresponding author upon reasonable request."

## Study approval

This study protocol was reviewed and approved by ethics committee on human research by Al Azhar faculty of medicine (IRB 00012367-00-00-025). All methods were performed in accordance with the relevant guidelines and regulations.
